# The Naples Prognostic Score May Predict No Reflow in Patients Undergoing Percutaneous Coronary Intervention for Saphenous Vein Graft Stenosis

**DOI:** 10.3390/diagnostics16050714

**Published:** 2026-02-27

**Authors:** Kadri Murat Gürses, Hüseyin Tezcan, Mustafa Bilal Özbay, Adnan Karaibrahimoglu, Çağrı Yayla, Halil Özalp, Muhammed Ulvi Yalçın, Abdullah Tunçez, Yasin Özen

**Affiliations:** 1 Medical Faculty Hospital, Department of Cardiology, Selçuk University, Konya 42130, Türkiye; 2 Department of Medicine, Penn Medicine Princeton Medical Center, Plainsboro, NJ 08536, USA; mbozbaymd@gmail.com; 3 Biostatistics & Medical İnformatics Deptartment, Medical School, Süleyman Demirel University, Isparta 32260, Türkiye; 4 Ankara City Hospital, Department of Cardiology, Ankara 06800, Türkiye

**Keywords:** no-reflow phenomenon, saphenous vein grafts, Naples Prognostic Score, thrombolysis in myocardial infarction score

## Abstract

**Background/Objectives:** No-reflow phenomenon (NRP) is a frequent and clinically relevant complication during percutaneous coronary intervention (PCI) of saphenous vein grafts (SVGs). The Naples Prognostic Score (NPS), a composite index reflecting systemic inflammation and nutritional status, may help identify patients at increased risk before the procedure. We investigated whether NPS predicts NRP in patients undergoing PCI/percutaneous transluminal coronary angioplasty (PTCA) for SVG stenosis. **Methods:** In this retrospective multicenter observational study, consecutive post-coronary artery bypass grafting patients undergoing PCI/PTCA for SVG stenosis were analyzed. NRP was defined as post-procedural thrombolysis in myocardial infarction (TIMI) flow grade <3 in the absence of dissection, residual stenosis, or vasospasm. NPS (0–4) was calculated from serum albumin, total cholesterol, neutrophil-to-lymphocyte ratio, and lymphocyte-to-monocyte ratio. Independent predictors of NRP were assessed using logistic regression, and discrimination was evaluated by receiver operating characteristic (ROC) analysis. **Results:** Among 252 patients, 55 (21.8%) developed NRP. NPS was significantly higher in the NRP group than in the normal-reflow group (2.61 ± 0.95 vs. 1.73 ± 0.95; *p* < 0.001). In multivariable analysis, NPS independently predicted NRP (per 1-point increase: odds ratio 2.577, 95% CI 1.428–5.384; *p* < 0.001 for univariate and 6.077, 95% CI 3.194–11.563; *p* < 0.001 for multivariate analysis), together with high thrombus burden (TIMI thrombus grades 4–5). NPS showed good discrimination for NRP (AUC 0.742; *p* < 0.001), with 75% sensitivity and 66% specificity at the optimal cut-off. **Conclusions:** NPS is a simple, readily available score that independently predicts angiographic no-reflow during SVG PCI and may aid preprocedural risk stratification and tailoring of preventive strategies.

## 1. Introduction

Coronary artery bypass grafting (CABG) is a revascularization technique used as an alternative to percutaneous transluminal coronary angioplasty (PTCA) or percutaneous coronary intervention (PCI) in patients with high SYNTAX (synergy between PCI and cardiac surgery) scores or in certain patient groups [1]. With approximately 400,000 CABG operations performed annually [2], saphenous vein grafts (SVGs) are the most commonly used grafts in CABG due to their easy accessibility in daily clinical practice and their lack of significant impact on venous circulation. Unfortunately, approximately 10% of SVGs become occluded in the first year following CABG [3]. SVG patency rates are halved during the first decade [4]. Subsequent SVG interventions also lead to numerous procedural problems, such as slow or absent retrograde flow and distal embolization [5]. No-reflow phenomenon (NRP) is a common complication of PTCA/PCI in SVGs. NRP is more common during the intervention of SVGs than with the intervention of native vessels, with an incidence of approximately 15% in recent publications [6,7,8]. Although NRP is frequently associated with different inflammatory indices in different cardiac patient groups, a perfect marker has not yet been discovered [9,10].

The Naples Prognostic Score (NPS), a new prognostic scoring system that has come of age in colorectal cancers in the last decade by Galizia et al., is a composite index based on four biomarkers that evaluates systemic inflammation and nutritional status [11,12]. Initially developed as a prognostic marker in oncology settings, the NPS has attracted attention for its potential role in predicting outcomes in cardiovascular disease [13,14,15]. Its ability to reflect the systemic inflammation and nutritional environment may provide valuable information about the risk of NRP in patients with SVG who undergo percutaneous intervention [16,17].

This study aims to assess the predictive usefulness of NPS in patients at increased risk of NRP who received percutaneous intervention for SVG. Through the analysis of this association, we intend to create a straightforward, bench-based evaluation instrument that assists physicians in identifying high-risk clients and enhancing their preprocedural management.

## 2. Patients and Methods

### 2.1. Population and Sample

This retrospective study included 331 consecutive patients who underwent coronary artery bypass grafting (CABG) and subsequent PCI/PTCA of the SVG at Ankara City Cardiovascular Hospital (previously named Ankara Yüksek İhtisas Training and Research Hospital) and Selçuk University Medical Faculty Hospital between February 2019 and July 2025. Fifty-six patients were eliminated from the trial due to severe renal disease, anemia, infection, and the use of steroids or anticoagulants. Patients receiving chronic oral anticoagulation were excluded; among included patients with atrial fibrillation, CHA_2_DS_2_-VASc scores were low, and anticoagulation was not clinically indicated. Although patients were enrolled from two centers, all procedures were performed by the same core interventional cardiology team, which migrated between institutions during the study period, resulting in largely standardized procedural techniques, device selection, and intraprocedural pharmacological strategies across sites.

**Figure 1:**
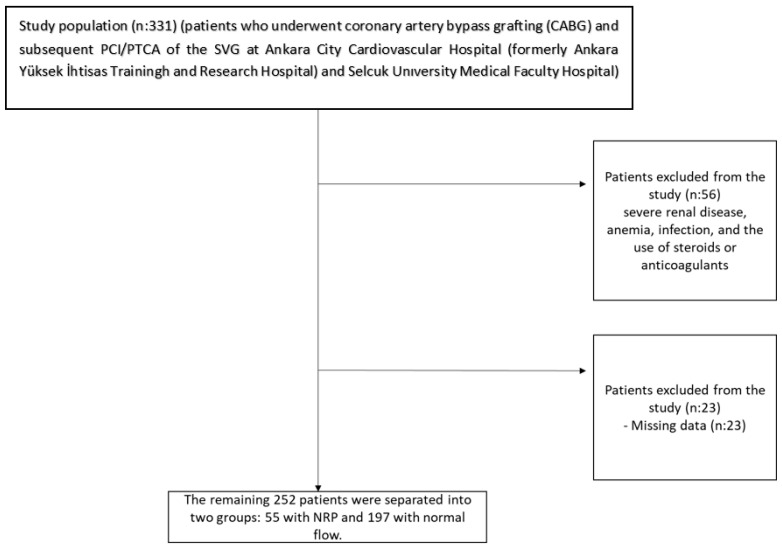
Flow chart of study population.

Twenty-three further cases were removed due to insufficient data (Figure 1).

Of the 252 included patients, 222 patients (88.8%) were enrolled from Ankara City Cardiovascular Hospital (formerly Ankara Yüksek İhtisas Training and Research Hospital) and 30 patients (12.2%) from Selçuk University Medical Faculty Hospital. The remaining 252 patients were separated into two groups: 55 with NRP and 197 with normal flow. The study protocol was approved by the local institutional ethics committee of Selçuk University Faculty of Medicine (Approval no: 2025/550) and conducted in accordance with the Declaration of Helsinki.

### 2.2. No-Reflow Phenomenon

NRP is described as insufficient myocardial tissue perfusion following a brief period of ischemia in the absence of mechanical obstruction, such as dissection, spasm, or thrombosis in the epicardial artery [7,18]. The concept of NRP encompasses the phenomenon of slow flow as well as the complete loss of coronary flow [18]. Numerous mechanisms for NRP have been implicated in the literature, including cellular edema [19], ischemia–reperfusion injury, microvascular spasm and microembolisms [20]. While NRP is an independent predictor of in-hospital mortality, its long-term effects include increased heart failure, mortality, and the incidence of major cardiac events [21,22]. Because there are no specifically developed treatment options for NRP, it poses a significant challenge for interventional cardiologists. Current procedural and pharmacological strategies have limited success in preventing NRP and managing it once it occurs [23]. Procedural strategies used to prevent NRP include direct stenting without predilatation and the use of short stents, embolic protection devices, and excimer lasers, while pharmacological strategies include the use of adenosine, nicardipine, and nitroprusside in the distal bed [24]. For the purposes of this study, NRP was defined as a post-operative coronary flow (TIMI) flow grade of less than 3 without evidence of dissection, stenosis, or vasospasm [25]. The presence of NRP was decided by the common consensus of the two primary operators of the study. If there was a discrepancy between these two operators, a third operator was referred for the final decision.

### 2.3. Naples Prognostic Score

The NPS score was calculated using four biomarkers, and each parameter was assigned a score of 0 or 1. Patients were scored from 0 to 4 based on this score. Serum albumin (≥4 g/dL scored as 0; <4 g/dL scored as 1), Total cholesterol (TC) (≥180 mg/dL scored as 0; <180 mg/dL scored as 1), Neutrophil-to-lymphocyte ratio (NLR) (≤2.96 scored as 0; >2.96 scored as 1), Lymphocyte-to-monocyte ratio (LMR) (>4.44 scored as 0; ≤4.44 scored as 1) [11]. Those with an NPS score of (0–2) were classified as the low NPS group, and those with a NPS score of (3–4) were classified as the high NPS group.

### 2.4. Statistical Analysis

The statistical analyses of the study were performed by SPSS 27.0 (IBM Inc, Armonk, NY, USA) software. The descriptive statistics were presented as mean ± SD for continuous variables, and frequency (percentage) for categorical variables. Numerical characteristics were compared between no-reflow groups by Student *t*-test, and the Mann–Whitney U test where necessary for non-normal distribution. The relation between the study groups and the categorical variables was assessed using the Chi-square test. ROC analysis was performed for some biochemical ratios and the Naples score, and the diagnostic ratios were calculated according to cut-off values. Univariate and multivariate binary logistic regression models were established for the no-reflow group. The multivariable logistic regression analysis was performed to assess the independent association between the NPS and the occurrence of NRP after SVG intervention, rather than to develop or validate a comprehensive predictive model or to formally evaluate incremental model performance. To avoid redundancy and overfitting, the NPS was entered into multivariable models as a composite variable, and its individual component biomarkers were not simultaneously included. Candidate variables for multivariable analysis were prespecified based on prior literature and biological plausibility, and the number of covariates was intentionally limited to preserve model parsimony and reduce the risk of overfitting. A *p* < 0.05 value was considered a statistically significant result for 5% type-I error.

## 3. Result

A total of 252 patients who underwent CABG and subsequently underwent PCI/PTCA of the SVG for a valid indication were included in the study. Of these, 21.8% had NRP, and the remainder had normal reflow with unaffected coronary flow. Demographic, clinical, and laboratory characteristics of the patients with and without NRP were compared (Table 1).

**Figure 2:**
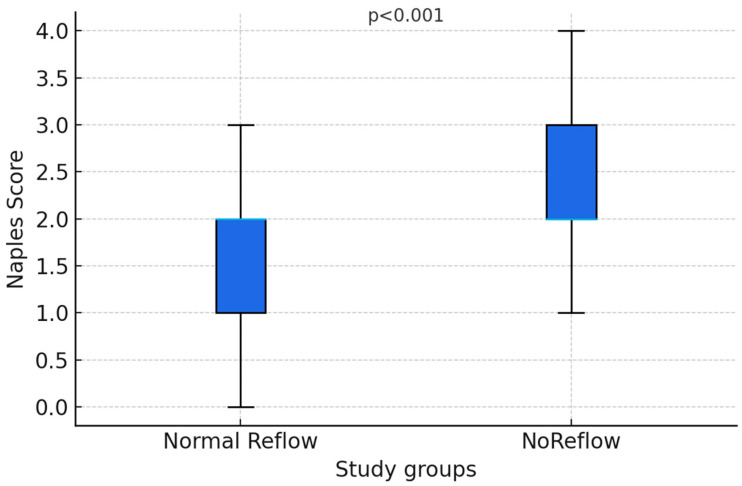
Naples score values according to reflow groups.

There was no difference between the two groups in terms of mean age and gender (*p* = 0.510 and *p* = 0.400, respectively). The mean ages of the NRP and normal reflow groups were calculated as 65.4 ± 10.1 and 66.5 ± 10.2 years, respectively. Compared to 81.1% of the NRP group, 75.6% of the normal reflow group consisted of males. On the other hand, the STEMI rate was higher in the NRP group (*p* = 0.021) compared to the angiography centers. Diabetes mellitus was more common in the NRP group (*p* < 0.001), but no statistically significant difference was observed in other comorbidities and medical treatments (Table 1). The Naples score was significantly higher in the no-reflow group (2.61 ± 0.95) than in the reflow group (1.73 ± 0.95) (*p* < 0.001) (Figure 2) (Table 1).

In laboratory tests, a statistically significant difference was observed between the values of Glucose [163.98 ± 66.11 vs. 139.69 ± 65.22 mg/dL, *p* = 0.019], Neutrophil [6.62 ± 2.82 vs. 5.23 ± 1.78 10^3^/mm^3^, *p* = 0.001], Lymphocyte [1.81 ± 0.75 vs. 2.32 ± 0.83 10^3^/mm^3^, *p* < 0.001], Monocytes [0.70 ± 0.34 vs. 0.57 ± 0.20 10^3^/mm^3^, *p* = 0.007]; No significant differences were observed between other biomarkers such as albumin, total cholesterol, hemoglobin, platelet LDL-C, HDL-C, creatinine, first troponin, and CRP (Table 1). Furthermore, there was no difference between the groups in terms of left ventricular ejection fraction (Table 1).

Among the periprocedural outcomes, stent length was significantly higher in the NRP group than in the normal reflow group [28.31 ± 15.01 mm vs. 23.17 ± 13.28 mm, *p* = 0.008]. There was also a significant difference between the groups in terms of TIMI score. Accordingly, 50% of patients who developed NRP had a Grade 5 thrombus score (*p <* 0.001) (Table 2).

The saphenous graft origin used was most frequently in the RCA (*p* = 0.029). The number of grafts, the number of stents, or the presence or absence of drug-eluting stents did not differ between the groups. NRP was observed to be statistically significantly more frequent among the patient groups with TIMI grade 4–5 scores (*p* < 0.001). Regarding clinical outcomes, Killip classification and length of hospital stay were statistically significantly higher in the NRP group (*p* = 0.003 and *p* = 0.008, respectively). Among the Naples score groups, a higher proportion of Group-2 patients was observed in the no-reflow group (*p* < 0.001) (Table 2).

In the univariate logistic regression analysis, the TIMI score of high grade (i.e., Grades 4 and 5) (OR: 3.891, 95% CI: 1.234–12.345, *p* = 0.020) had a significant increasing effect on no-reflow. In addition, Naples score (OR: 2.577, 95% CI: 1.428–5.384, *p <* 0.001) has a high risk of no-reflow status (2.57 times). Other clinical parameters, including diabetes mellitus *(p* = 0.103), heart failure *(p* = 0.454), Killip classification (*p* = 0.597), and glucose levels (*p* = 0.773), were not statistically significantly associated with the outcome.

To identify independent predictors, variables with significant associations were included in a multivariate logistic regression model. The results of the multivariate analysis demonstrated that both the TIMI score (OR: 5.512, 95% CI: 1.851–16.393, *p* = 0.002) and the Naples score (OR: 6.077, 95% CI: 3.194–11.563, *p <* 0.001) remained independent predictors of no-reflow, with the Naples score exhibiting the highest odds for predicting the development of the phenomenon (Table 3).

**Figure 3:**
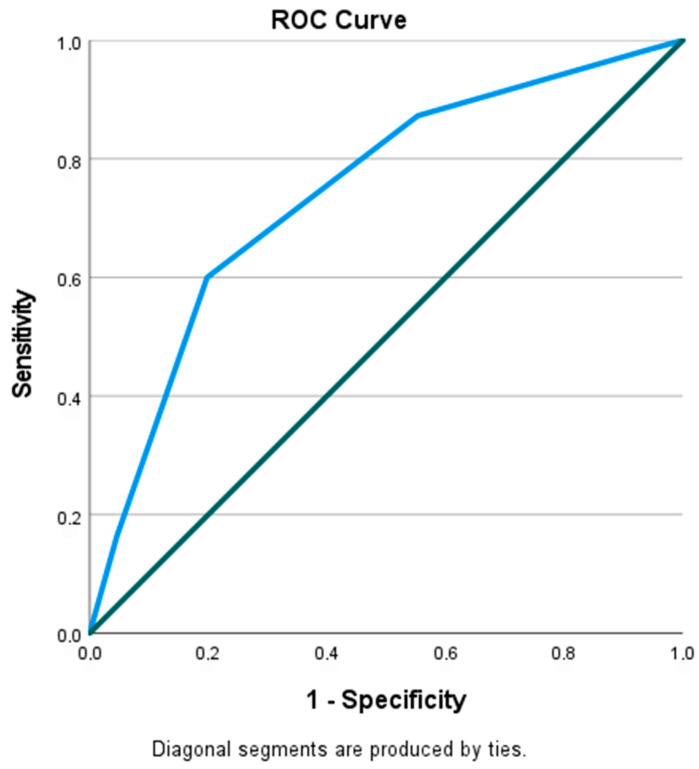
ROC curve of Naples Score for reflow groups (AUC = 0.742; *p* < 0.001).

The receiver operating characteristic (ROC) curve analysis revealed that the optimal cutoff value of the Naples prognostic score for predicting the development of NRP in patients undergoing PTCA/PCI of the SVG was 2.50. The diagnostic ratios, sensitivity, and specificity are 75% and 66%, respectively [Area under the curve (AUC): 0.742; *p*-value < 0.001]. (Figure 3).

## 4. Discussion

This study demonstrated that NPS is independently associated with NRP, a bothersome complication, in patients undergoing percutaneous SVG intervention. Our study is the only study in the literature to test the predictive value of NPS for NRP, as it included both patients with ACS and those undergoing elective SVG intervention.

In recent years, advancements in coronary imaging have enabled more patients with coronary artery disease (CAD) to be diagnosed in a timely manner, enabling earlier intervention. In parallel with this, increased CABG surgery and the anatomical characteristics of SVGs, resulting in occlusion rates reaching 15% in the first year and approximately half within the first decade, have led to a greater frequency of both elective and acute graft occlusions and a greater need for percutaneous SVG intervention [26,27]. However, interventions in CABG patients have some disadvantages, such as longer operating times, more complex, undesirable SVG anatomy and a more dense thrombus environment, use of more opaque compared to native PCI, and incompatibility of grafts and catheters [18]. Therefore, the incidence of certain complications is significantly higher during SVG procedures compared with native coronary arteries [4,28]. One of these complications, NRP, is a serious adverse event of PCI. It is one of the most common undesirable situations encountered during SVG interventions [29].

In the literature, NRP has been associated with heart failure and malignant arrhythmias and has been shown to be associated with in-hospital mortality for this reason [30]. The association of NRP with mortality after post-interventional cardiovascular interventions and the lack of a therapeutic approach have led cardiologists to further understand the pathogenesis of NRP. A number of studies have been conducted in this context. Some of these studies, as mentioned above, have addressed the mechanisms that may cause NRP. Previous studies have demonstrated several important determinants of NRP, including diabetes mellitus (DM), advanced age, male gender, prior history of CABG, elevated troponin I levels, elevated creatinine levels, Killip class ≥ 2, and lesion length [31,32]. Ashraf et al. mentioned that pre-procedure TIMI flow grade may be a risk factor for NRP [31]. Similarly, we found that the higher incidence of NRP was observed in patients with high TIMI flow grade.

NRP is observed in approximately 5% of all patients undergoing PCI, while this rate is observed in around 18% of SVG interventions [29,33]. We believe that SVG interventions have unique risk factors for NRP, and that the inclusion of only patients who underwent SVG intervention in this study enhances the value of this study. Indeed, while many underlying mechanisms have been discussed, no definitive underlying mechanism has been identified. Previous studies have shown that increased local inflammatory activity in the microcirculation increases the risk of no-reflow [4,34]. This has created a need for operators to develop a scoring system that can correlate multiple inflammatory markers and identify patients at risk of this poor outcome during the procedure. While Wang et al. identified the neutrophil count as an independent predictor of NRP in patients presenting with STEMI, Dogan et al. demonstrated that low lymphocyte counts may be associated with NRP [10,35]. Later, Kocas et al. argued that NLR is associated with high TIMI frame count [36]. In a previous publication, we showed that systemic immune-inflammation index (SII) was associated with NRP in the patient group who underwent PCI to the SVG due to ACS [24]. The search for a scoring system with this prognostic value has continued, leading to the association of NRP with different scoring systems in different patient groups in recent years. Liu et al. correlated the hemoglobin, albumin, lymphocyte, and platelet (HALP) score with NRP in STEMI patients [37], while Mo et al. correlated NRP with the HbA1c/C peptide ratio in STEMI patients [38]. Gene Fedai et al. correlated the CALLY index, calculated using inflammatory markers C-reactive protein (CRP), albumin, and lymphocytes, with NRP in STEMI patients [39]. However, we found only one study showing the relationship between NPS, which reflects the inflammatory and nutritional status of patients, and the development of no-reflow after SVG PCI, which is associated with cardiovascular diseases [40].

NPS was first described by Galizia et al. in 2017, and its relationship with prognosis was demonstrated in patients with gastrointestinal malignancies [11]. Although initially a focus of research in various cancer types, NPS has been associated with poor outcomes in many cardiac patient groups in recent years [41,42,43]. Zhu et al. showed that NPS is a reliable tool for predicting 30-day mortality in acute pulmonary embolism [44]. Demirci et al. demonstrated that NPS is reliable for all-cause mortality in long-term follow-up of transcatheter aortic valve replacement patients [45]. Erdogan et al. demonstrated that NPS is an independent predictor of mortality in STEMI patients during in-hospital and 15-month follow-up [41]. Gitmez et al. showed that this score predicts 1-year mortality in Non-STEMI patients [42]. In another study, in which the authors of this study also participated, we found an association between collateral development and NPS in patients with chronic total occlusion [46]. In our meta-analysis published in early 2026, which included 7 studies, we found that high NPS in ACS patients was associated with poor clinical outcomes [47]. Finally, Yılmaz et al. demonstrated that NPS was a predictor of NRP when PCI was performed on SVG in a patient group similar to our single-center, retrospective study [40]. In addition, it was stated in this study that other factors that pose a risk for NRP may be stent diameter and post-dilatation [40]. While the patient group with NRP in this study was 16.2%, this rate constituted 21.8% of the patients in our study. Our study had some advantages over this study. Some of these include recruiting patients from at least two centers, assessing periprocedural outcomes, and including both ACS and elective patients. Furthermore, Yılmaz et al. noted that they did not evaluate periprocedural data in their study [40]. In our study, unlike Yılmaz et al., we found that stent diameter did not pose a risk for NRP [40]. Additionally, in our study, the number, type, and length of stents did not pose a risk for NRP. Because we did not have access to the patients’ postdilatation data, we were unable to compare these findings.

The potential role of NPS in predicting the prognosis of many cardiovascular diseases reported in the literature may be related to its inclusion of these inflammatory and nutritional biomarkers. Indeed, our findings demonstrate a significant association between the NPS and the occurrence of NRP, a vexing complication for cardiology operators when dealing with SVG. Each one-point increase in NPS was associated with a 2.57-fold (or 6.07-fold) higher odds of NRP during PCI. Calculating this score is quite straightforward, and our findings suggest that calculating it before SVG PCI may be helpful in identifying individuals at high risk.

These findings should be interpreted in light of the observational design and do not imply causality. Rather than serving as a mechanistic mediator of no-reflow, NPS should be viewed as a prognostic marker reflecting global disease burden, systemic inflammation, nutritional status, and overall clinical vulnerability. By capturing reduced physiological reserve, higher NPS identifies patients at increased susceptibility to procedural complications during complex SVG interventions. Accordingly, the clinical value of NPS lies in risk stratification and pre-procedural identification of high-risk patients, while mechanistic links to microvascular dysfunction require confirmation in prospective studies.

Despite the NPS remaining independently associated with NRP after adjustment for established clinical and angiographic predictors, including thrombus burden, its incremental predictive value beyond conventional risk factors was not formally assessed. Changes in model discrimination, calibration, or reclassification were not evaluated; therefore, the additive prognostic contribution of NPS cannot be definitively established. NPS may partly capture risk already reflected by traditional variables such as acute coronary syndrome presentation, DM, or lesion complexity. Accordingly, NPS should be considered a complementary marker integrating systemic inflammatory and nutritional risk rather than a stand-alone predictor. Future studies using formal model-performance analyses are needed to determine whether incorporating NPS meaningfully improves risk prediction and clinical decision-making in saphenous vein graft interventions.

NRP was defined as post-procedural TIMI flow grade < 3, a widely used angiographic criterion in native coronary and SVG interventions [48]. However, this definition does not fully distinguish epicardial flow limitation from true microvascular no-reflow and may reflect heterogeneous mechanisms. The absence of complementary microvascular indices limits biological specificity and mechanistic interpretation of the association between the NPS and NRP. Accordingly, these findings should be interpreted in terms of angiographic and clinical risk stratification rather than direct microvascular pathology, and future studies incorporating detailed microvascular assessment are warranted.

An additional consideration is potential temporal confounding in patients presenting with ACS. Components of the NPS, particularly leukocyte-based inflammatory indices, may be acutely influenced by the ischemic event and stress response, complicating interpretation as markers associated with no-reflow [49]. Accordingly, in ACS presentations, NPS likely reflects a composite of baseline systemic vulnerability and acute-phase inflammation. While this supports its role in real-time risk stratification, it limits mechanistic inference, and future studies with stratified or interaction analyses are needed to determine whether the association with no-reflow differs across clinical contexts.

Because several components of the NPS, such as neutrophil and lymphocyte counts, have been individually associated with no-reflow, potential construct redundancy and collinearity warrant consideration [50]. In this study, NPS was modeled as a composite index rather than alongside its constituent variables, consistent with its design as an integrated measure of systemic inflammatory and nutritional status and to minimize redundancy in multivariable analyses. Accordingly, individual components were not simultaneously included in the regression model. Although this approach supports parsimony and clinical interpretability, formal collinearity diagnostics were not performed, and the incremental prognostic value of NPS beyond its individual biomarkers cannot be fully established. Future studies directly comparing NPS with its components using formal collinearity and model-performance analyses are warranted.

The cut-off values used to construct the NPS were originally derived from oncology and nutrition–inflammation research settings [51], and their biological relevance and distribution may differ in patients with advanced CAD undergoing CABG and saphenous vein graft intervention. In cardiovascular populations, chronic low-grade inflammation, metabolic comorbidities, and prior revascularization may influence baseline levels of albumin, lipid profiles, and leukocyte-derived indices, potentially altering the discriminatory performance of individual thresholds [52,53]. In the present study, established cut-offs were applied to maintain consistency with prior literature and to evaluate the performance of the composite score rather than to recalibrate individual components. Accordingly, these findings should be interpreted as supporting the prognostic utility of NPS as an integrated marker, while future studies are needed to determine whether population-specific cut-offs or alternative weighting of components may further refine risk stratification in PCI cohorts.

Although our findings demonstrate a significant association, several limitations should be acknowledged. First, it should be noted that the study design was cross-sectional and retrospective. Although data were collected from two different centers, they cannot be generalized to the general population due to the unequal number of patients. Second, balloon angioplasty performed on patients before or after stent placement was not included in the analysis. Other studies have suggested that this may contribute to the development of NRP [40,54], which may influence the results. Finally, the sample size was relatively small. Therefore, larger-scale studies are needed on this patient population undergoing SVG interventions; these interventions account for less than 10% of all percutaneous interventions, but they are valuable.

Emerging evidence suggests that pharmacological therapies from the cardiometabolic field may favorably modulate myocardial perfusion and microvascular function in patients with myocardial infarction. In particular, glucagon-like peptide-1 receptor agonists (GLP-1RAs) have been associated with improvements in myocardial perfusion and ischemia-related outcomes [55], raising the possibility that incorporation of cardiometabolic treatment status or responsiveness could be considered in the future development and refinement of prognostic scores for PCI.

## 5. Conclusions

This study demonstrates that the NPS, which can be easily calculated from blood values and provides significant convenience for operators, is an independent predictor of NRP development in patients undergoing SVG intervention. Our study is also the only study in the literature to examine all patient groups undergoing SVG intervention, whether they were undergoing ACS or elective procedures. Furthermore, patients with high TIMI flow grades are also at risk for NRP.

## 6. Limitations

Several limitations of this study should be acknowledged. The retrospective and observational design precludes causal inference. Despite operator-level standardization, residual center- or period-related effects related to institutional workflows, temporal changes in practice, or patient case mix cannot be fully excluded and represent an inherent limitation of the retrospective multicenter design. In addition, the absence of formal incremental model performance analyses represents an important limitation, as the additive prognostic value of the NPS beyond established risk factors could not be formally assessed. Although data were derived from two tertiary referral centers, the overall sample size was relatively modest, which may limit statistical power and generalizability to broader populations undergoing SVG intervention.

Detailed procedural variables, including pre- and post-dilatation strategies and the use of distal embolic protection devices, were not uniformly available across centers and therefore could not be incorporated into the analyses. Inflammatory biomarkers were assessed at a single time point, which may not adequately reflect dynamic inflammatory changes over time. In patients presenting with acute coronary syndromes, admission inflammatory parameters used to calculate the NPS may have been influenced by the acute ischemic event itself, introducing temporal ambiguity that could not be fully addressed without stratified or interaction analyses.

No-reflow was defined solely by post-procedural TIMI flow grade, reflecting epicardial coronary flow and lacking complementary microvascular perfusion indices. Consequently, the ability to distinguish epicardial flow impairment from true microvascular dysfunction was limited, and the findings should be interpreted as angiographic rather than tissue-level no-reflow.

Because patients receiving chronic oral anticoagulation were excluded, individuals with atrial fibrillation or other high thrombotic-risk conditions may have been under-represented, introducing potential selection bias and limiting generalizability. In the final cohort, the few patients with atrial fibrillation had low CHA_2_DS_2_-VASc scores and therefore did not have a guideline-based indication for long-term anticoagulation.

Given the limited number of no-reflow events, multivariable analyses may be susceptible to overfitting, and the omission of some clinically relevant covariates—partly due to incomplete data availability—may have introduced residual confounding. In addition, operator-driven procedural modifications may have contributed to confounding by indication, further limiting causal interpretation of the observed associations. In addition, the cut-off values used to define individual NPS components were derived primarily from non-cardiovascular populations and may not be optimally calibrated for patients undergoing SVG intervention. Finally, there are some missing data regarding categorical variables, but these are not high enough to affect statistical analyses.

Prospective, large-scale studies incorporating comprehensive procedural data, serial biomarker measurements, and advanced modeling approaches are warranted to validate and extend these findings.

## Figures and Tables

**Table 1:** Basal demographic and laboratory characteristics.

	No-Reflow (*N* = 55)	Normal Reflow (*N* = 197)	*p*
	Mean ± SD (Median; Min–Max)		
**Demographic characteristics**			
Age	65.49 ± 10.10	66.52 ± 10.29	0.510
Gender			0.400
Male	43 (81.1) ^+^	149 (75.6)	
Female	10 (18.9) ^+^	48 (24.4)	
**Application**			0.021 *
Non-ACS	21 (38.2)	100 (50.8)	
Nonstemi	24 (43.6)	84 (42.6)	
Stemi	10 (18.2)	13 (6.6)	
**Comorbidites**			
Diabetes mellitus	37 (67.3)	79 (40.1)	<0.001 *
Heart failure	19 (34.5)	48 (24.4)	0.090
Smoking	20 (36.3)	53 (26.9)	0.163
iCVA	2 (3.6)	5 (2.5)	0.666
Rhythm			0.456
Afib	0 (0.0)	2 (1.0)	
NSR	54 (100.0)	193 (99.0)	
**Treatments**			
GIIb-IIIa inhibitors	18 (32.7)	17 (8.6)	0.144
Acetyl salicylic acid	51 (92.7)	190 (96.4)	0.234
Clopidogrel	43 (78.2)	164 (83.2)	0.437
Ticagrelor	5 (9.0)	17 (8.6)	0.901
Prasugrel	2 (3.6)	5 (2.5)	0.655
RAAS inhibitors	44 (80.0)	170 (86.2)	0.286
Beta-blockers	50 (91.0)	191 (96.9)	0.160
Aldactone	7 (12.7)	16 (8.1)	0.257
**Biochemical tests**			
Glucose (mg/dL)	163.98 ± 66.11	139.69 ± 65.22	0.019 *
Albumin (g/dL)	4.17 ± 0.43	4.13 ± 0.34	0.603
Total cholesterol (mg/dL)	163.98 ± 57.15	168.64 ± 55.76	0.586
Hemoglobin (g/dL)	13.62 ± 1.72	13.38 ± 1.77	0.378
WBC (10^3^/mm^3^)	9.25 ± 2.59	8.44 ± 2.65	0.056
Platelet (10^3^/mm^3^)	223,717.39 ± 63,909.54	227,430.37 ± 59,277.32	0.714
Neutrophil (10^3^/mm^3^)	6.62 ± 2.82	5.23 ± 1.78	0.001 *
Lymphocyte (10^3^/mm^3^)	1.81 ± 0.75	2.32 ± 0.83	<0.001 *
Monocytes (10^3^/m^3^)	0.70 ± 0.34	0.57 ± 0.20	0.007 *
Hematocrit (%)	40.65 ± 6.96	40.64 ± 5.62	0.991
MPV (fL)	9.94 ± 1.48	9.66 ± 1.31	0.241
LDL-C (mg/dL)	125.90 ± 54.89	113.41 ± 54.91	0.154
Triglyceride (mg/dL)	196.94 ± 194.70	185.84 ± 173.64	0.698
HDL-C (mg/dL)	45.08 ± 32.58	41.59 ± 11.16	0.239
Urea (mg/dL)	44.58 ± 22.75	42.35 ± 17.56	0.520
Creatinin (mg/dL)	1.06± 0.34	1.04 ± 0.28	0.678
Total protein (g/dL)	8.47 ± 9.98	7.97 ± 8.04	0.770
Peak CK MB (ng/mL)	44.52 ± 49.49	27.91 ± 29.26	0.093
First Troponin * (ng/L)	10.12 ± 28.29	6.75 ± 53.20	0.723
Sedimentation	28.46 ± 26.49	20.73 ± 21.02	0.169
CRP (mg/dL)	15.51 ± 28.34	9.31 ± 18.78	0.178
LVEF (%) ± SD	43.91 ± 9.99	47.02 ± 10.45	0.065
Naples Score	2.61 ± 0.95 (3; 0–4)	1.73 ± 0.95 (2; 0–4)	<0.001 **

*: significant at 0.05 level according to Student’s *t*-test. **: significant at 0.05 level according to the Mann–Whitney U test. * At admission. ^+^ Percentages are calculated using the number of patients with available data for each variable. Gender data were missing for two patients in the no-reflow group^.^ ACS, acute coronary syndrome; Afib, atrial fibrillation; STEMI, ST elevation myocardial infarction; NSTEMI, non-ST elevation myocardial infarction; NSR, normal sinus rhythm; RAAS, renin–angiotensin–aldosterone system; iCVA, ischaemic cerebrovascular accident; CK MB, creatine kinase myocardial band; LDL-C, low-density lipoprotein cholesterol; HDL-C, high-density lipoprotein cholesterol; CRP, C-reactive protein; WBC, white blood cell; MPV, mean platelet volume; LVEF, left ventricular ejection fraction; SD, standard deviation.

**Table 2:** Angiographic and procedural characteristics and clinical outcomes.

	No-Reflow (*N* = 55)	Normal Reflow (*N* = 197)	*p*
Location of the saphenous graft			0.029 *
Rca	33 (60.0)	79 (40.1)	
Diagonal	3 (5.5)	26 (13.2)	
Cx	7 (12.7)	26 (13.2)	
Lad	3 (5.5)	11 (5.6)	
Om	9 (16.3)	54 (27.4)	
Ima	0 (0.0)	1 (0.5)	
**Procedural data**			
Number of grafts	2.64 ± 0.59 (3; 1–4)	2.77 ± 0.75 (3; 1–5)	0.313
Number of stents	1.27 ± 0.65 (1; 0–3)	1.22 ± 0.52 (1; 0–3)	0.340
Stent type			0.184
DES	16 (37.2)	91 (48.4)	
BMS	27 (62.8)	97 (51.6)	
Stent length (mm)	28.31 ± 15.01 (24; 8–83)	23.17 ± 13.28 (20; 8–89)	0.008 **
Stent diameter (mm)	3.36 ± 0.55 (3.5; 2.5–4)	3.20 ± 0.49 (3; 2.2–4.5)	0.257
TIMI score			<0.001 *
Grade 0	0 (0.0)	12 (6.3)	
Grade 1	2 (4.0)	5 (2.6)	
Grade 2	0 (0.0)	15 (7.9)	
Grade 3	3 (6.0)	46 (24.2)	
Grade 4	20 (40.0)	90 (47.4)	
Grade 5	25 (50.0)	22 (11.6)	
**Clinical outcomes**			
Killip classification			0.003 *
Killip 1	45 (81.8)	182 (92.4)	
Killip 2	4 (7.3)	12 (6.1)	
Killip 3	4 (7.3)	3 (1.5)	
Killip 4	2 (3.6)	0 (0.0)	
Length of hospital stay	3.81 ± 2.38 (3;1–10)	3.49 ± 3.71 (2; 0–22)	0.008 **
Additional intervention at admission	8 (14.8)	19 (9.7)	0.289
**Cardiac events**			0.696
ACS	2 (3.6)	7 (3.6)	
Stent thrombosis	2 (3.6)	1 (0.5)	
None	47 (85.5)	171(87.2)	
All	4 (7.3)	17 (8.7)	
**Application Form**			0.039 *
Non-ACS	21 (38.2)	100 (50.8)	
ACS	32 (58.2) 2 (3.6)	97 (49.2)	
Naples groups			<0.001 *
Group 1	22 (40.0)	158 (80.2)	
Group 2	33 (60.0)	39 (19.8)	

*: significant at the 0.05 level according to the Chi-square test. ACS, acute coronary syndrome; RCA, right coronary artery; Cx, circumflex artery; LAD, left anterior descending artery; OM, obtuse marginal artery; IMA, intermediate artery; DES, drug-eluting stent; BMS, bare-metal stent; TIMI, thrombolysis in myocardial infarction. ** Significant at the 0.01 level according to the Chi-square test.

**Table 3:** Univariate and Multivariate Logistic Regression Models.

Univariate Model				Multivariate Model		
Coefficient	Beta	*p*	OR (95% CI)	Beta	*p*	OR (95% CI)
Diabetes mellitus	0.892	0.103	2.440 (0.834–7.143)			
Heart failure	0.350	0.454	1.420 (0.567–3.552)			
TIMI score	1.359	0.020 *	3.891 (1.234–12.345)	1.707	0.002 *	5.512 (1.851–16.393)
Killip classification		0.597				
Killip (1)	−2.297	0.889	0.100 (0.004–1.980)			
Killip (2-3-4)	−1.510	0.992	0.220 (0.008–1.916)			
Naples Score	0.947	<0.001 *	2.577 (1.428–5.384)	1.804	<0.001 *	6.077 (3.194–11.563)
Glucose	−0.001	0.773	0.999 (0.989–1.008)			
Constant	19.271	0.999	2,341,196	−0.524	0.251	

*: Significant at 0.05 level according to binary logistic regression, OR: Odds ratio, CI: Confidence Interval, TIMI, thrombolysis in myocardial infarction.

## Data Availability

The data presented in this study are available upon reasonable request from the corresponding author. The data are not publicly available due to institutional and ethical restrictions.
